# Effects of Water Extracts of *Graptopetalum paraguayense* on Blood Pressure, Fasting Glucose, and Lipid Profiles of Subjects with Metabolic Syndrome

**DOI:** 10.1155/2013/809234

**Published:** 2013-11-25

**Authors:** Chi-Hua Yen, Shu-Ju Chen, Jen-Tzu Liu, Yu-Fen Tseng, Ping-Ting Lin

**Affiliations:** ^1^Department of Family and Community Medicine, Chung Shan Medical University Hospital, Taichung 40201, Taiwan; ^2^School of Medicine, Chung Shan Medical University, Taichung 40201, Taiwan; ^3^Center for Education and Research on Geriatrics and Gerontology, Chung Shan Medical University, Taichung 40201, Taiwan; ^4^Department of Health Food, Chung Chou University of Science and Technology, Changhua 51003, Taiwan; ^5^School of Nutrition, Chung Shan Medical University, Taichung 40201, Taiwan; ^6^Department of Nutrition, Chung Shan Medical University Hospital, Taichung 40201, Taiwan

## Abstract

This study was aimed to investigate the effects of water extracts of *Graptopetalum paraguayense* (WGP, 4 g/d) on blood pressure, blood glucose level, and lipid profiles in subjects with metabolic syndrome (MS). Participants with MS (*n* = 54) were randomly assigned to the placebo (*n* = 28) and WGP groups (*n* = 26), and the intervention was administered for 12 weeks. Systolic blood pressure (SBP), diastolic blood pressure (DBP), fasting glucose (FG), lipid profiles (total cholesterol (TC), triglyceride (TG), low density lipoprotein cholesterol (LDL-C), and high density lipoprotein (HDL-C)), and antioxidant enzymes activities (catalase (CAT), superoxide dismutase (SOD), and glutathione peroxidase (GPx)) were measured. Forty-two subjects completed the study (placebo, *n* = 19; WGP, *n* = 23). FG, SBP, and LDL-C levels were significantly lower and HDL-C level and antioxidant enzymes activities (CAT and SOD) were significantly higher after WGP supplementation. Blood pressure, FG, and lipid profiles were significantly correlated with antioxidant enzymes activities after supplementation (*P* < 0.05). The present study demonstrated a significant reduction in blood pressure, blood glucose, and lipid profiles and an increase in antioxidant enzymes activities in subjects with MS after WGP supplementation. Taken together, the antioxidative capacity of WGP might exert a beneficial effect on MS. This trial is registered with ClinicalTrials.gov NCT01463748.

## 1. Introduction

Metabolic syndrome (MS) represents a collection of physiological and anthropometric abnormalities [1] and is recognized as a significant risk factor for cardiovascular disease and type II diabetes [2]. The National Health and Nutrition Examination Surveys (NHANES) indicated that more than 30% of the adult population in the USA suffered from MS [3–5]. In Taiwan, reports from the Nutrition and Health Survey in Taiwan (NAHSIT) indicated that the prevalence of MS in adults (≥18 y) was 20% during 1993–1996 and increased to 28.5% during 2005–2008 [6]. The characteristics of MS, including insulin resistance, type II diabetes, hypertension, dyslipidemia, and visceral obesity, may increase oxidative stress [7–9] and reduce antioxidant defenses [10–12]. Increases in oxidative stress contribute to impaired vascular function, thrombosis, and atherosclerosis that ultimately result in vascular disease [13]. 

*Graptopetalum paraguayense* E. Walther is a popular traditional Chinese herbal medicine that belongs to the *Crassulaceae* family. Several biological activities of *Graptopetalum paraguayense* have been reported, including antityrosine [14], radical-scavenging [15], antihypertensive [16, 17], and hepatoprotective effects [18–20], in addition to protection against brain injury in ischemic rats [21]. Most studies have investigated the functions of *Graptopetalum paraguayense* in cell or animals models [14–21]. To our knowledge, Lin et al. [22] conducted the only clinical study where subjects with hypercholesterolemia consumed *Graptopetalum paraguayense* as a serving of vegetable (100 g/d) for 8 weeks and found a significant decrease in the level of oxidative stress but no change in lipid profiles. These results may be because of the suboptimal *Graptopetalum paraguayense *dosage and short intervention duration. Therefore, this study was aimed to examine the effects of a 12-week supplementation with water extracts of *Graptopetalum paraguayense *(WGP) on blood pressure, fasting glucose (FG), and lipid profiles of subjects with MS. 

## 2. Materials and Methods

### 2.1. Participants

Individuals with MS were recruited from the Department of Family and Community Medicine at the Chung Shan Medical University Hospital in Taiwan. The inclusion criteria for MS in adults were based on the guidelines of the Administration of Health Promotion, Ministry of Health and Welfare, Taiwan (2007). Subjects were considered to have MS if they had three of the following five characteristics: (1) abdominal obesity (waist circumference ≥90 cm in men and ≥80 cm in women), (2) impaired FG (≥5.6 mmol/L), (3) hypertriglyceridemia (triglyceride (TG) ≥1.7 mmol/L), (4) low high density lipoprotein cholesterol (HDL-C) <1.0 mmol/L in men and <1.3 mmol/L in women, and (5) increased blood pressure (systolic blood pressure (SBP) ≥130 mmHg and diastolic blood pressure (DBP) ≥85 mmHg). Subjects using antidiabetic, antihypertensive, and lipid-lowering medications were considered to have elevated FG, elevated blood pressure, and dyslipidemia, respectively. The diagnostic criteria for MS in Taiwan are in accordance with the National Cholesterol Education Program Adult Treatment Panel III (ATP III, 2001), International Diabetes Federation (IDF, 2005), and the American Heart Association/National Heart, Lung, and Blood Institute (AHA/NHLBI, 2005) criteria [1, 23, 24]. Therefore, the diagnostic criteria for MS in Taiwan are similar with these international criteria. This study was approved by the Institutional Review Board of the Chung Shan Medical Hospital in Taiwan, and the written informed consent was obtained from each subject. The trial was registered at ClinicalTrials.gov (no. NCT01463748).

### 2.2. Administration of Water Extracts of *Graptopetalum paraguayense* (WGP)

Fresh *Graptopetalum paraguayense* E. Walther was purchased from a local organic farm (TOPF0024, Yunlin County, Taiwan). The dose (4 g/d) of the *Graptopetalum paraguayense *administered was chosen according to the report by Lin et al. [22]. The water extraction rate of *Graptopetalum paraguayense* was 4% [14], and as a result, 100 g of *Graptopetalum paraguayense *provides 4 g WGP. The safety of the WGP dose was assessed by Chung et al. [25], and WGP is considered safe with a no observed adverse effect level (NOAEL) of 1.0 g/kg body weight in rats, which is equivalent to a daily dose of 0.16 g/kg of body weight in humans. The WGP and placebo (starch) capsules were prepared by the Gold Chia Fong Biotech Co., Ltd. (Taichung, Taiwan). 

### 2.3. Study Design

**Figure 1:**
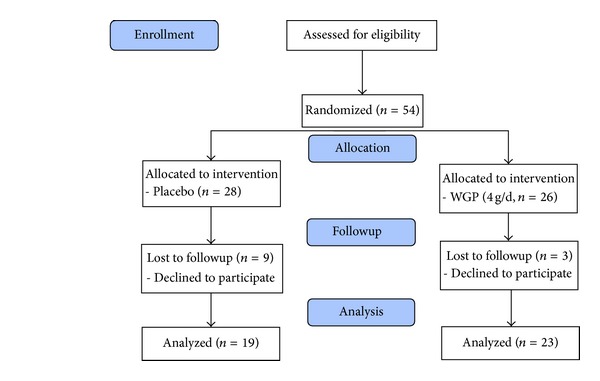
CONSORT scheme. WGP: water extracts of *Graptopetalum paraguayense*.

We conducted a single-blind randomized, parallel, placebo-controlled study. The sampling and trial schemes are summarized in Figure 1. With a sample size calculation, we expected that the change in the levels of antioxidant enzymes activities would be 5.0 ± 7.0 units/mg of protein after the WGP intervention; therefore, the desired power was set at 0.8 to detect a true effect and at an *α* value equal to 0.05 with a minimal sample size of 18 subjects in each intervention group. We enrolled 54 subjects with MS in this study that were randomly assigned to the placebo (*n* = 28) or WGP (*n* = 26) group. The female subjects in this study were postmenopausal women who were not receiving hormone therapy. The intervention was administered for 12 weeks, and the subjects were instructed to take eight capsules daily (WGP 4 g/d and 0.5 g/capsule). To monitor compliance, the researchers reminded subjects to check the capsule bag every 4 weeks to confirm that the bag was empty. The age; blood pressure; and smoking, drinking, and exercise habits of all subjects were recorded. Body weight and height were measured; the body mass index (kg/m^2^) was then calculated.

### 2.4. Biochemical Measurement

Fasting venous blood samples (15 mL) were collected to estimate the hematological parameters. Blood specimens were collected in vacutainer tubes without EDTA as an anticoagulant. Serum was prepared and then frozen (−80°C) for storage until analysis. The quantitative measurements of FG and blood lipid profiles [i.e., total cholesterol (TC), triglyceride (TG), LDL-C, and HDL-C] were measured using an automated biochemical analyzer (Hitachi-7180E, Tokyo, Japan). Red blood cells (RBCs) were diluted with 25x sodium phosphate buffer for superoxide dismutase (SOD) and glutathione peroxidase (GPx) measurements and with 250x sodium phosphate buffer for catalase (CAT) measurement. The methods for measuring CAT, SOD, and GPx were previously described [26–28]. The protein content of plasma and RBCs was determined based on the biuret reaction using a BCA kit (Thermo, Rockford, IL, USA). The antioxidant enzymes activities were expressed as units/mg of protein. All analyses were performed in duplicate.

### 2.5. Statistical Analyses

The data were analyzed using SigmaPlot software (version 12.0, Systat, San Jose, CA, USA). The normality of the distribution of the variables was evaluated using the Shapiro-Wilk test. Differences in subject demographics and hematological measurements between the placebo and WGP groups were analyzed by Student's *t*-test or the Mann-Whitney rank sum test. The paired *t*-test or Wilcoxon signed rank test was used to analyze the data within each group before (baseline) and after intervention (week 12). For categorical response variables, differences between the two groups were assessed by the Chi-square test or Fisher's exact test. To examine the correlations of blood pressure, FG, lipid profiles, and antioxidant enzymes activities after supplementation, the Pearson product moment correlation or Spearman rank order correlation was used. The data are expressed as the means ± standard deviations (SD). The results were considered statistically significant at *P* < 0.05.

## 3. Results

### 3.1. Characteristics of the Participants

Forty-two subjects completed the study (placebo, *n* = 19; WGP, *n* = 23).  Table 1 presents the demographic data and health characteristics of the subjects. There were no significant differences between the two groups with respect to age; anthropometric measurements; and the frequency of smoking, drinking, or exercise at baseline.

### 3.2. Blood Pressure, FG, and Lipid Profiles after WGP Supplementation

**Figure 2:**
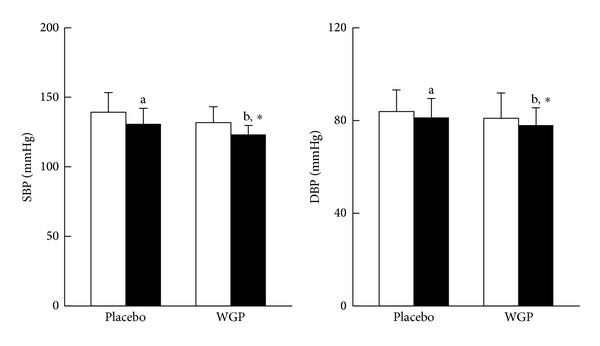
Blood pressure measurements. Data are means ± SD. □ Week 0, ■ Week 12. *Values were significantly different after intervention within the group, *P* < 0.05. ^a,b^Values with different superscripts were significantly different between two groups, *P* < 0.05. DBP: diastolic blood pressure; SBP: systolic blood pressure; WGP: water extracts of *Graptopetalum paraguayense*.

**Figure 3:**
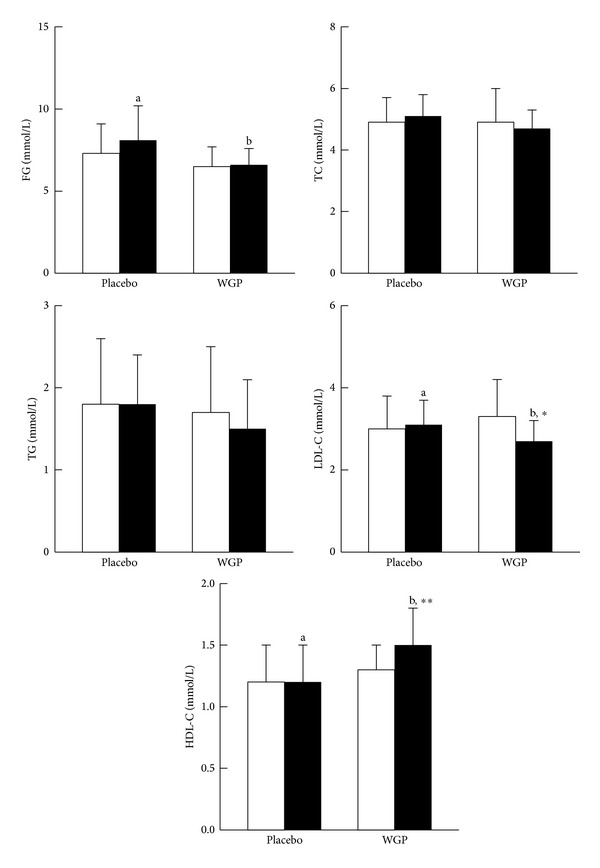
Fasting glucose level and blood lipid profiles. Data are means ± SD. □ Week 0, ■ Week 12. *Values were significantly different after intervention within the group; *P = 0.06; **P = 0.01. ^a,b^Values with different superscripts were significantly different between two groups, *P* < 0.05. FG: fasting glucose; HDL-C: high density lipoprotein cholesterol; LDL-C: low density lipoprotein cholesterol; TC: total cholesterol; TG: triglyceride; WGP: water extracts of *Graptopetalum paraguayense*.

Blood pressure, FG, and lipid profiles measurements are presented in Figures 2 and 3. The levels of SBP (*P* < 0.01) and LDL-C (*P* = 0.06) were significantly decreased, while HDL-C was significantly increased (*P* = 0.01) after WGP supplementation. The subjects in the WGP group had significantly lower SBP (*P* = 0.01), FG (*P* = 0.04), TC (*P* = 0.08), TG (*P* = 0.07), and LDL-C (*P* = 0.04) levels and higher HDL-C (*P* = 0.01) level than those in the placebo group at week 12.

### 3.3. Antioxidant Enzymes Activities after WGP Supplementation

**Figure 4:**
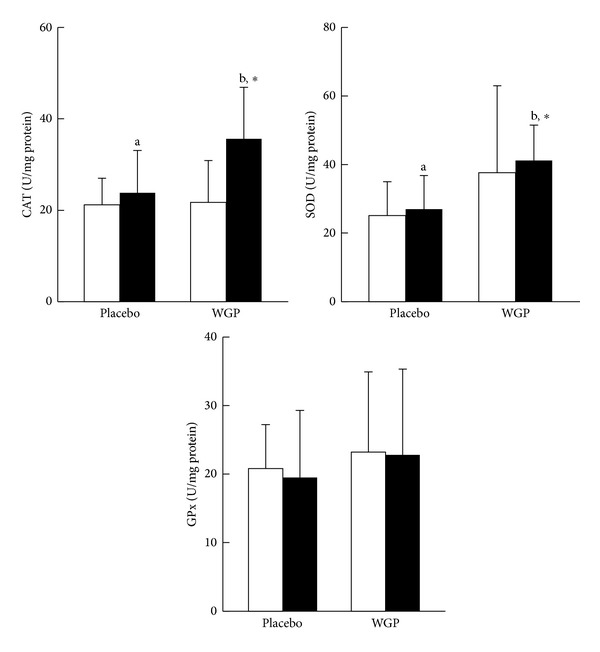
Antioxidant enzymes activities. Data are means ± SD. □ Week 0, ■ Week 12. *Values were significantly different after intervention within the group, *P* < 0.05. ^a, b^Values with different superscripts were significantly different between two groups, *P* < 0.05. CAT: catalase; GPx: glutathione peroxidase; SOD: superoxide dismutase; WGP: water extracts of *Graptopetalum paraguayense*.

The antioxidant enzymes activities are presented in Figure 4. The activities of CAT (*P* < 0.01) and SOD (*P* = 0.02) were significantly increased after WGP supplementation. The subjects in the WGP group had significantly higher CAT and SOD activities than those in the placebo group at week 12 (*P* < 0.01).

### 3.4. Correlations between Blood Pressure, FG, Lipid Profiles, and Antioxidant Enzymes Activities after WGP Supplementation

The correlations between blood pressure, FG, lipid profiles, and antioxidant enzymes activities after 12 weeks of supplementation are presented in Table 2. After 12 weeks of supplementation, the antioxidant enzymes activities were significantly negatively correlated with the SBP, DBP, and FG level and positively correlated with HDL-C level (*P* < 0.05). The change in antioxidant enzymes activities was significantly correlated with the change in DBP and lipid profiles levels (TC, TG, and HDL-C, *P* < 0.05).

### 3.5. Adverse Events

There were no clinically significant changes in the subjects' vital signs, serum chemical values, or hematological values, and there were no serious adverse events in either group.

## 4. Discussion

This is the first clinical study to report a statistically significant link between the components of MS and antioxidant status in MS subjects treated with WGP. In the present study, we observed that the characteristics of MS (blood pressure, FG, and lipid profiles) were significantly correlated with the antioxidant status (Table 2) after 12 weeks of WGP supplementation. The antioxidant capacity of *Graptopetalum paraguayense *has been demonstrated in both *in vitro* and *in vivo* studies [14–22]. Oxidative stress is thought to play an important role in the development of MS [9]. In general, individuals with MS are typically abdominally obese. Obesity is an oxidative burden that may lead to the reduction of antioxidant enzymes activities [29]. Antioxidant enzymes are the first line of defense against reactive oxygen species (ROS) and lead to a decrease in their activities [12, 30]. In the present study, the activities of CAT and SOD were significantly increased by 51% and 28%, respectively, after 12 weeks of WGP supplementation. Plant phenol and polyphenolic compounds, such as flavonoids and anthocyanins, are widely distributed in natural herb and spice extracts, and they possess significant antioxidant activities [30, 31]. Chung et al. reported that the total phenolic compounds and anthocyanins in *Graptopetalum paraguayense* extracts ranged from 11.0 to 34.0 mg/g and from 0.03 to 1.29 *μ*mol/g, respectively; these compounds may increase the radical-scavenging ability [15]. As a result, WGP may contribute to the first line of antioxidation, leading to increases in antioxidant status.

 Hypertension and hyperglycemia induce the activation of oxidative stress pathways and mediate the development of cardiovascular disease and diabetes [2, 32]. In this study, we treated subjects with MS using WGP at dose of 4 g daily for 12 weeks; SBP and FG levels were significantly decreased by 8% and 3%, respectively, after treatment. Similar results were also found in animals models [17, 33]. For hypertension treatment, the inhibition of angiotensin converting enzyme (ACE) is a major modern therapeutic approach. The extracts of *Graptopetalum paraguayense* have demonstrated an antihypertension effect because of their inhibitory effects on ACE activity [17]. With respect to glucose regulation, pancreatic *β*-cells are sensitive to ROS and the peroxidation of *β*-cells may amplify the glucotoxicity and lipotoxicity effects of hyperglycemia [34]. Therefore, antioxidants, such as WGP, may exhibit a protective effect against oxidative stress by neutralizing ROS and improving insulin sensitivity in subjects with MS.

 Previous studies on lipid metabolism have demonstrated that human serum paraoxonase (PON 1) may inhibit the oxidation of LDL-C and exclusively bind to HDL-C [35–37]. After 12 weeks of WGP supplementation, LDL-C level was significantly decreased by 16% and HDL-C level was significantly increased by 10%. *Graptopetalum paraguayense* could modulate the lipid metabolism by increasing PON 1 gene expression [38]. PON 1 can hydrolyze lipid peroxides in oxidized lipoproteins and atherosclerotic lesions [39]. In addition, *Graptopetalum paraguayense* might contain flavonoids and dietary fiber, which are important mediators in the regulation of lipid metabolism in MS and obesity [40, 41].

Lin et al. [22] reported that *Graptopetalum paraguayense* was related to side effects including diarrhea and oral ulcers. However, no subjects reported these side effects in the present study, possibly because the *Graptopetalum paraguayense *was administered as an oral supplement in capsule form. There were no clinically significant changes in the subjects' vital signs, serum chemical values, or hematological values, and no subject withdrew because of adverse events. Thus, WGP at a dose of 4 g/d is safe for individuals with MS. 

The population size is a limitation of the present study. The number of participants was small, although we recruited more subjects than expected. Large studies are needed to establish the beneficial effects of WGP supplementation. 

In conclusion, subjects suffering from MS may exhibit a higher level of oxidative stress. The present study demonstrated a significant reduction in blood pressure, blood glucose, and lipid profiles and an increase in the antioxidant enzymes activities in subjects with MS after WGP supplementation. WGP might exert a beneficial effect on MS related to its antioxidative capacity. 

## Figures and Tables

**Table 1:** Subjects characteristics.

	Placebo (*n* = 19)	WGP (*n* = 23)	*P* values
Male/female (*n*)	12/7	14/9	0.88
Age (y)	56.2 ± 9.7 (57.0)	50.8 ± 11.8 (52.0)	0.12
Waist circumference (cm)	100.2 ± 10.4 (100.0)	98.5 ± 12.3 (96.0)	0.62
Waist hip ratio	0.9 ± 0.1 (0.9)	0.9 ± 0.1 (0.9)	0.87
Body mass index (kg/m^2^)	29.4 ± 4.1 (29.1)	29.3 ± 4.7 (29.3)	0.80
Current smoker^1^, *n* (%)	3 (15.8%)	2 (8.7%)	0.64
Consuming alcohol^2^, *n* (%)	2 (10.5%)	2 (8.7%)	1.00
Exercise^3^, *n* (%)	11 (57.9%)	14 (61.9%)	0.90

Data are given as mean ± SD (median).

^1^Current smoker: individuals currently smoking one or more cigarettes per day.

^2^Consuming alcohol: individuals regularly drinking one or more alcoholic beverages per day.

^3^Exercise: individuals exercising regularly at least three times every week.

WGP: water extracts of* Graptopetalum paraguayense*.

**Table 2:** Correlations (*r* ^1^) between blood pressure, fasting glucose, lipid profiles, and antioxidant enzymes activities after 12 weeks of supplementation.

	CAT (U/mg protein)		SOD (U/mg protein)		GPx (U/mg protein)	
	Week 12	Δ12–0^2^	Week 12	Δ12–0	Week 12	Δ12–0
SBP (mmHg)						
Week 12	−0.41*		−0.10		−0.26*	
Δ12–0		−0.07		0.09		−0.19
DBP (mmHg)						
Week 12	−0.25*		−0.17*		−0.28*	
Δ12–0		−0.04		−0.11		−0.25*
FG (mmol/L)						
Week 12	−0.01*		−0.01*		−0.01*	
Δ12–0		9.44 × 10^−3^		1.11 × 10^−3^		3.89 × 10^−3^
TC (mmol/L)						
Week 12	−4.14 × 10^−3^		0.00		−2.59 × 10^−4^	
Δ12–0		−7.25 × 10^−3∗^		−1.55 × 10^−3^		−6.99 × 10^−3∗^
TG (mmol/L)						
Week 12	−1.70 × 10^−3^		−2.26 × 10^−3^		−1.36 × 10^−3^	
Δ12–0		−3.16 × 10^−3∗^		−4.41 × 10^−3∗^		−4.52 × 10^−3∗^
LDL-C (mmol/L)						
Week 12	−3.77 × 10^−3^		−4.14 × 10^−3^		−3.63 × 10^−3^	
Δ12–0		1.04 × 10^−3^		3.89 × 10^−3^		−7.77 × 10^−4^
HDL-C (mmol/L)						
Week 12	0.01*		7.25 × 10^−3∗^		1.55 × 10^−3^	
Δ12–0		0.00		0.01*		3.89 × 10^−3^

^1^Correlation coefficient.

^2^Δ12–0, changes from week 0 to week 12.

*P < 0.05.

CAT: catalase; FG: fasting glucose; GPx: glutathionine peroxidase; HDL-C: high density lipoprotein-cholesterol; LDL-C: low density lipoprotein-cholesterol; SOD: superoxide dismutase; TC: total cholesterol; TG: triglyceride; WGP: water extracts of *Graptopetalum paraguayense*.
